# A taxonomic framework for emerging groups of ecologically important marine gammaproteobacteria based on the reconstruction of evolutionary relationships using genome-scale data

**DOI:** 10.3389/fmicb.2015.00281

**Published:** 2015-04-09

**Authors:** Stefan Spring, Carmen Scheuner, Markus Göker, Hans-Peter Klenk

**Affiliations:** ^1^Department Microorganisms, Leibniz Institute DSMZ – German Collection of Microorganisms and Cell CulturesBraunschweig, Germany; ^2^School of Biology, Newcastle UniversityNewcastle upon Tyne, UK

**Keywords:** bacterioplankton, trophic guilds, oligotroph, *K*-selection, phylogenomics

## Abstract

In recent years a large number of isolates were obtained from saline environments that are phylogenetically related to distinct clades of oligotrophic marine gammaproteobacteria, which were originally identified in seawater samples using cultivation independent methods and are characterized by high seasonal abundances in coastal environments. To date a sound taxonomic framework for the classification of these ecologically important isolates and related species in accordance with their evolutionary relationships is missing. In this study we demonstrate that a reliable allocation of members of the oligotrophic marine gammaproteobacteria (OMG) group and related species to higher taxonomic ranks is possible by phylogenetic analyses of whole proteomes but also of the RNA polymerase beta subunit, whereas phylogenetic reconstructions based on 16S rRNA genes alone resulted in unstable tree topologies with only insignificant bootstrap support. The identified clades could be correlated with distinct phenotypic traits illustrating an adaptation to common environmental factors in their evolutionary history. Genome wide gene-content analyses revealed the existence of two distinct ecological guilds within the analyzed lineage of marine gammaproteobacteria which can be distinguished by their trophic strategies. Based on our results a novel order within the class *Gammaproteobacteria* is proposed, which is designated *Cellvibrionales* ord. nov. and comprises the five novel families *Cellvibrionaceae* fam. nov., *Halieaceae* fam. nov., *Microbulbiferaceae* fam. nov., *Porticoccaceae* fam. nov., and *Spongiibacteraceae* fam. nov.

## Introduction

For several years an increasing effort in the characterization of bacteria from marine environments led to the isolation of a large number of gammaproteobacteria that belong to phylogenetic groups originally defined by 16S rRNA gene sequences retrieved from environmental samples. Many of these novel isolates can be allocated to the oligotrophic marine gammaproteobacteria (OMG) group as defined by Cho and Giovannoni (2004). The five distinct clades of the OMG group (OM60/NOR5, OM182, SAR92, KI89A, and BD1-7) are phylogenetically related, but do not form a robust monophyletic lineage in 16S rRNA gene based trees. Consequently, it seems likely that at least some of these clades have evolved independently from each other as adaptation to oligotrophic conditions encountered in seawater. Within the OMG group members of the OM60/NOR5 and SAR92 clades are of special interest, because cultivation-independent surveys have shown that these bacteria can reach a high seasonal abundance especially in coastal environments (Stingl et al., 2007; Yan et al., 2009). In addition, genome analyses revealed the potential of harvesting light for mixotrophic growth in several members of the OMG group, which made these isolates interesting study objects for further research (e.g., Stingl et al., 2007; Spring and Riedel, 2013). Genes involved in the complex pathway of aerobic anoxygenic photosynthesis were so far only found among members of the OM60/NOR5 clade (Spring et al., 2013, 2014), whereas genes for the synthesis of proteorhodopsin, a light-driven proton pump, seems to be more widely spread and were identified in the genomes of representatives of the SAR92 (Courties et al., 2014), BD1-7 (Oh et al., 2010a), and OM60/NOR5 clades (Jang et al., 2011b).

Currently, most of the novel gammaproteobacterial strains that were obtained by high-throughput cultivation approaches and have been affiliated to the OMG group were not described as novel species and thus remain unclassified (Cho and Giovannoni, 2004). On the other hand, some isolates that were recently described as novel species were either not allocated to higher taxonomic ranks at the family and order level or their assignment to higher order taxa is inconsistent with the phylogenetic relationships among the *Gammaproteobacteria* as shown for example in recent trees (although without branch support) distributed by the all-species living tree project (Yarza et al., 2008; release LTPs115, http://www.arb-silva.de/projects/living-tree/). This leads to a confusing classification scheme of environmental important marine gammaproteobacteria that is not in line with the presumable evolutionary relationships. Part of the confusion may have been caused by a close relationship of recently isolated strains affiliated to the OMG group with species regarded as non-oligotrophic and belonging to established genera like *Cellvibrio* and *Microbulbifer*, which have been originally affiliated to the families *Pseudomonadaceae* and *Alteromonadaceae*, respectively. Consequently, unclassified members of the OMG group are intermingled with species that have been affiliated with several different orders of *Gammaproteobacteria* leading to a classification scheme comprising various paraphyletic taxa.

However, the definition of meaningful higher taxonomic units comprising members of environmental important clades of the OMG group and related species could be of great value for the cultivation-independent analyses of microbial populations in marine environments (e.g., Ruff et al., 2014). Possible applications include the automatic classification of ribosomal RNA gene amplicon reads obtained by high-throughput sequencing using curated databases (e.g., SILVAngs, https://www.arb-silva.de/ngs/#about:) or the correlation of group-specific oligonucleotide probes used for *in situ* hybridization experiments with the phenotypic characteristics associated with well-defined microbial taxa. For this reason the aim of this study was to define a taxonomic framework for major clades of the OMG group and related described species that is based on the unveiled evolutionary relationships among these bacteria and thus less arbitrary than the current classification.

It was shown previously, that the identification of clades within the *Gammaproteobacteria* is difficult based solely on comparative analyses of 16S rRNA gene sequences due to the limited number of informative characters and the slow evolutionary rate of this molecule. Especially, if a large number of closely related species is compared the obtained branching patterns are often unstable, as indicated by a low bootstrap support of distinct tree topologies (e.g., Yamamoto and Harayama, 1998; Cho and Giovannoni, 2004; Spring et al., 2013). In contrast, housekeeping genes encoding large proteins seem to be more promising for phylogenetic analyses because calculations are based on a larger dataset and analyses of protein sequences are less prone to the nucleotide compositional bias seen in 16S rRNA (Wu and Eisen, 2008; Mulet et al., 2010). Among the known housekeeping proteins the RNA polymerase beta subunit encoded by the *rpoB* gene seems to be the most promising candidate as universal phylogenetic marker, especially for the classification of gammaproteobacteria. Initially, the potential application of the RpoB protein as reliable phylogenetic marker was demonstrated for bacteria by Rowland et al. (1993) and for Archaea by Klenk and Zillig (1994). Later it was shown by Mollet et al. (1997) that phylogenetic trees of *Enterobacteriaceae* based on *rpoB* genes were more compatible with their accepted classification than those obtained with 16S rRNA. Several further studies have demonstrated that *rpoB* genes can be used as a good proxy of the respective genome sequences, which is indicated for example by a strong correlation between the G + C content of the *rpoB* gene and the corresponding genome (Adékambi et al., 2009). Above all, among several tested housekeeping proteins the phylogenetic signal of the RpoB protein shows the highest correlation with the corresponding amino acid identity values of the respective genomes (Konstantinidis and Tiedje, 2005).

With the increasing number of genome sequences that became available for members of the OMG group and related taxa, especially in the course of large-scale sequencing projects (Wu et al., 2009; Yooseph et al., 2010; Kyrpides et al., 2014), a more advanced reconstruction of evolutionary relationships based on genome-scale data became possible (Delsuc et al., 2005; Klenk and Göker, 2010), thereby avoiding stochastic errors related to the use of single genes (Wu and Eisen, 2008). Initially, this approach was questioned by an assumed high incidence of lateral gene transfer between distantly related taxa of prokaryotes, which would lead to a substantial loss of phylogenetic signal inherited by genome sequences (e.g., Gogarten et al., 2002). However, in the case of *Gammaproteobacteria* it was clearly shown that a high phylogenetic concordance exists among a large number of gene families, hence it is possible to infer reliable and robust tree topologies from genome-scale data (Lerat et al., 2003). In following studies the superiority of using proteome data (i.e., concatenated protein alignments) over single genes for the phylogenetic resolution of *Gammaproteobacteria* was asserted (Wu and Eisen, 2008; Williams et al., 2010).

Based on the reasons given above we used in the present study datasets based on the complete RpoB protein and whole proteomes besides a thorough analysis of the available 16S rRNA gene sequences in order to unravel the evolutionary relationships among members of the OMG group and related gammaproteobacteria.

As a result of our study the novel order *Cellvibrionales* is proposed to accommodate recently isolated marine gammaproteobacteria belonging to the OM60/NOR5, SAR92, and BD1-7 clades of the OMG group as well as related described species with a so far absent or misleading classification into higher order taxa.

## Materials and methods

### Phylogenetic analyses based on single molecules

Comprehensive datasets of 16S rRNA gene sequences of *Gammaproteobacteria* comprising cultured and uncultured members of the OMG group were derived from the alignment included in the non-redundant version of the SILVA Ref database (Quast et al., 2013; SSU Ref NR 99 release 119). Firstly, based on the manually curated trees included in the SILVA Ref NR 119 (http://www.arb-silva.de/download/arb-files/) and LTPs 115 (http://www.arb-silva.de/projects/living-tree/) databases a range of relevant type strains with a recognizable relationship to major clades of the OMG group were determined. Then, additional outgroup sequences were selected that represent type genera of all major families and orders within the class *Gammaproteobacteria*. Whenever possible only 16S rRNA gene sequences of type strains of type species with validly published names as given in the List of Prokaryotic Names with Standing in Nomenclature (LPSN; Parte, 2014) were used in order to avoid the inclusion of accidentally misclassified strains. The number of outgroup sequences representing a distinct family was limited to a maximum number of six to keep the calculation time required for bootstrap analyses practicable. The accession numbers of the used 16S rRNA gene sequences are given in Supplementary Table S1.

Based on the obtained 16S rRNA gene phylogeny a range of full-length RpoB protein sequences of genome-sequenced strains were selected that covers isolates and described species affiliated to clades of the OMG group as well as representative type species of *Pseudomonadales, Oceanospirillales, Alteromonadales*, and *Enterobacteriaceae* as outgroup sequences. RpoB protein sequences were obtained from GenBank or Uniprot and aligned using the ClustalW algorithm implemented in the ARB package. The accession numbers of the used RpoB proteins are given in Table 1.

**Table 1:** **Dataset of RpoB protein sequences and genome sequences used for the reconstruction of RpoB-based trees or supermatrix trees based on whole proteomes**.

**Strain**	**RpoB protein acc. no**.	**Genome acc. no**.	**Classification^a^**
***CELLVIBRIONALES* ord. nov**.			
*Cellvibrio japonicus* Ueda107^T^	B3PK30	CP000934	*Cellvibrionaceae*
*Cellvibrio* sp. BR	I3I669	AICM00000000	*Cellvibrionaceae*
*Gilvimarinus chinensis* [T] DSM 19667^T^	WP_020211074	ARIX00000000	*Cellvibrionaceae*
*Marinimicrobium agarilyticum* DSM 16975^T^	WP_027330681	AUHU00000000	*Cellvibrionaceae*
*Saccharophagus degradans* [T] 2-40^T^	Q21M93	CP000282	*Cellvibrionaceae*
*Simiduia agarivorans* [T] DSM 21679^T^	K4KJ11	CP003746	*Cellvibrionaceae*
*Teredinibacter turnerae* [T] T7902^T^	WP_018274091	ARAH00000000	*Cellvibrionaceae*
*Congregibacter litoralis* [T] KT71^T^	A4A6A0	AAOA00000000	*Halieaceae*
*Congregibacter* sp. NOR5-3	B8KNT7	ACCX00000000	*Halieaceae*
Gammaproteobacterium HTCC2080	A0Z129	AAVV00000000	*Halieaceae*
Gammaproteobacterium HTCC2148	B7S356	ABXQ00000000	*Halieaceae*
Gammaproteobacterium HIMB55	H3NW15	AGIF00000000	*Halieaceae*
Gammaproteobacterium IMCC3088	F3L4H4	AEIG00000000	*Halieaceae*
*Haliea salexigens* [T] DSM 19537^T^	J9Z3P3	AUHJ00000000	*Halieaceae*
*Luminiphilus syltensis* [T] NOR5-1B^T^	B8KWF4	ACCY00000000	*Halieaceae*
*Pseudohaliea rubra* [T] DSM 19751^T^	R4J8J3	AUVB00000000	*Halieaceae*
*Microbulbifer agarilyticus* S89	WP_010130329	AFPJ00000000	*Microbulbiferaceae*
*Microbulbifer variabilis* ATCC 700307^T^	WP_020411122	AQYJ00000000	*Microbulbiferaceae*
Gammaproteobacterium HTCC2207	Q1YNY6	AAPI00000000	*Porticoccaceae*
Gammaproteobacterium MOLA455	W2UEW1	AZIN00000000	*Porticoccaceae*
“*Porticoccus hydrocarbonoclasticus”* MCTG13d	U740DRAFT_0413	JQMM00000000	*Porticoccaceae*
*Dasania marina* [T] DSM 21967^T^	WP_026244726	ARDZ00000000	*Spongiibacteraceae*
Gammaproteobacterium BDW918	I2JM08	AJMK00000000	*Spongiibacteraceae*
Gammaproteobacterium HTCC2143	A0YHK1	AAVT00000000	*Spongiibacteraceae*
*Spongiibacter marinus* (=*Melitea salexigens*) [T] DSM 19753	WP_027875072	AULP00000000	*Spongiibacteraceae*
*Spongiibacter tropicus* DSM 19543^T^	WP_022960717	ATUS00000000	*Spongiibacteraceae*
**OUTGROUP REFERENCES**			
*Alcanivorax borkumensis* [T] SK2^T^	Q0VSM2	AM286690	*Alcanivoracaceae*
*Alcanivorax pacificus* W11-5^T^	K2HC73	AJGP00000000	*Alcanivoracaceae*
*Alcanivorax dieselolei* B5^T^	K0CEN5	CP003466	*Alcanivoracaceae*
*Alteromonas macleodii* [T] ATCC 27126^T^	J9YDH2	CP003841	*Alteromonadaceae*
*Glaciecola punicea* [T] ACAM 611^T^	H5T848	BAET00000000	*Alteromonadaceae*
*Glaciecola nitratireducens* FR1064^T^	G4QDW1	CP003060	*Alteromonadaceae*
*Paraglaciecola polaris* LMG 21857^T^	K6ZKT0	BAER00000000	*Alteromonadaceae*
*Salinimonas chungwhensis* [T] DSM 16280^T^	WP_026294703	ARJT00000000	*Alteromonadaceae*
*Enterobacter cloacae* subsp. *cloacae* [T] ATCC 13047 ^T^	D5CBL4	CP001918	*Enterobacteriaceae*
*Rahnella aquatilis* [T] CIP 78.65^T^	H2IVU9	CP003244	*Enterobacteriaceae*
*Xenorhabdus nematophila* [T] ATCC 19061^T^	D3VEF0	FN667742	*Enterobacteriaceae*
*Providencia alcalifaciens* [T] DSM 30120^T^	B6X9R3	ABXW00000000	*Enterobacteriaceae*
*Hahella chejuensis* [T] KCTC 2396^T^	Q2S905	CP000155	*Hahellaceae*
*Hahella ganghwensis* DSM 17046^T^	WP_020405297	AQXX00000000	*Hahellaceae*
*Halomonas elongata* [T] DSM 2581^T^	E1V5K0	FN869568	*Halomonadaceae*
*Halomonas zhanjiangensis* DSM 21076^T^	WP_018917740	ARIT00000000	*Halomonadaceae*
*Chromohalobacter salexigens* DSM 3043^T^	Q1R0I2	CP000285	*Halomonadaceae*
*Kushneria aurantia* [T] DSM 21353^T^	WP_026351855	ARNK00000000	*Halomonadaceae*
*Perlucidibaca piscinae* [T] DSM 21586^T^	WP_022957094	ATUE00000000	*Moraxellacea*
*Moraxella bovoculi* 237^T^	KDN25377	AOMT00000000	*Moraxellaceae*
*Psychrobacter arcticus* 273-4^T^	Q4FQH3	CP000082	*Moraxellaceae*
*Acinetobacter calcoaceticus* [T] DSM 30006^T^	N9DU07	AIEC00000000	*Moraxellaceae*
*Acinetobacter radioresistens* DSM 6976^T^	K6VIF1	APQF00000000	*Moraxellaceae*
*Oceanospirillum maris* subsp. *maris* DSM 6286^T^	WP_028304193	AUGW00000000	*Oceanospirillaceae*
*Oceanospirillum beijerinckii* subsp. *beijerinckii* DSM 7166^T^	WP_028301410	AULT00000000	*Oceanospirillaceae*
*Marinospirillum minutulum* [T] DSM 6287^T^	WP_027849030	AULO00000000	*Oceanospirillaceae*
*Pseudomonas aeruginosa* [T] PAO1	Q51561	AE004091	*Pseudomonadaceae*
*Pseudomonas sutzeri* ATCC 17588^T^	F8H9S9	CP002881	*Pseudomonadaceae*
*Pseudomonas syringae* pv. *tabaci* ATCC 11528	F3K5M0	AEAP00000000	*Pseudomonadaceae*
*Pseudomonas veronii* 1YdBTEX2	WP_017848798	AOUH00000000	*Pseudomonadaceae*
*Azotobacter vinelandii* ATCC BAA-1303	C1DKK5	CP001157	*Pseudomonadaceae*
*Saccharospirillum impatiens* [T] DSM 12546^T^	WP_028671302	AUIH00000000	“*Saccharospirillaceae*”
*Reinekea blandensis* MED297^T^	A4BF53	AAOE00000000	“*Saccharospirillaceae*”
**ROOT**			
*Magnetococcus marinus* [T] MC-1^T^	A0L5W6	CP000471	*Magnetococcaceae*

*The [T] after a species epithet indicates a type species; the superscript T following a strain designation a type strain*.

a *For members of the proposed order Cellvibrionales the classification of strains at family level is based on the results of this study, whereas the classification of the outgroup references has been adopted from the NCBI Taxonomy Database (http://www.ncbi.nlm.nih.gov/taxonomy)*.

Phylogenetic trees based on datasets of single sequences were reconstructed using programs implemented in the ARB software package (Ludwig et al., 2004), PAUP^*^ (Swofford, 2002), RAxML (Stamatakis, 2006), or TNT (Goloboff et al., 2008). No filter or weighting masks were applied to constrain the used positions of the alignment. Phylogenetic distances were calculated with the correction of Felsenstein for nucleotide sequences (Felsenstein, 1982) or with PAM for amino acid sequences, when the ARB neighbor-joining program was used. Maximum parsimony trees were inferred with PAUP^*^ and for the 16S rRNA gene data set, which was larger in terms of organisms, with TNT. Maximum likelihood trees were reconstructed using the RAxML maximum likelihood program (version 7.0.3) with the GTRGAMMA model for DNA and PROTCATLGF for the RpoB data, which was determined as optimal empirical model using RAxML. In each case the robustness of the tree topologies was evaluated by performing 1000 rounds of bootstrap replicates.

### Phylogenetic analyses based on genome-scale data

The selection of species used for phylogenetic analyses using genome-scale data was identical to the data set used for the RpoB protein trees. Total protein sequences of 59 strains belonging to the class *Gammaproteobacteria* and one outgroup taxon (*Magnetococcus marinus* MC-1^T^) were obtained from the IMG website (https://img.jgi.doe.gov/cgi-bin/er/main.cgi) with the exception of the proteomes of the strains MOLA455 and *Congregibacter litoralis* KT71^T^ which were obtained from the NCBI website (http://www.ncbi.nlm.nih.gov/genome). The accession numbers of all used genome sequences are given in Table 1. Genome sequences were phylogenetically investigated using the phylogenomics pipeline developed at the Leibniz Institute DSMZ including the software applications NCBI BLAST (Altschul et al., 1997), OrthoMCL (Li et al., 2003), MUSCLE (Edgar, 2004), RASCAL (Thompson et al., 2003), and GBLOCKS (Talavera and Castresana, 2007). This method was firstly described by Spring et al. (2010) and later successfully applied to the phylogenetic structuring of several unresolved taxonomic groups of prokaryotes (e.g., Anderson et al., 2011; Abt et al., 2012; Meier-Kolthoff et al., 2014a; Scheuner et al., 2014). In brief, clusters of orthologs were generated using OrthoMCL, inparalogs were removed, the remaining sequences were aligned with MUSCLE and filtered with RASCAL and GBLOCKS. Then, three different supermatrices were compiled: (i) Filtered alignments comprising at least four sequences were concatenated to form a supermatrix. (ii) The supermatrix was cleaned from relatively uninformative genes using MARE (Meusemann et al., 2010) under default values (except that deleting organisms was disallowed). (iii) Those alignments containing 60 sequences (one sequence per genome) were concatenated to form the core-genes matrix.

The OrthoMCL clusters were also converted to an ortholog-content matrix representing the presence or absence of a gene within a certain genome and clusters of orthologs. Further, clusters of homologous sequences were determined using a re-implementation of the TribeMCL algorithm (Enright et al., 2002), applying an *e*-value threshold of 10^−5^ and an MCL inflation parameter of 2.0. The clusters of homologs were converted to a gene-content matrix in analogy to the ortholog-content matrix.

Maximum likelihood and maximum-parsimony trees were inferred from each matrix with RAxML (Stamatakis, 2006) and PAUP^*^ (Swofford, 2002), respectively, as previously described (e.g., Spring et al., 2010).

## Results and discussion

### Evolutionary relationships between members of the OMG group and related species

#### Phylogeny based on 16S rRNA genes

Phylogenetic analyses were based on a comprehensive dataset of 200 nearly complete 16S rRNA gene sequences resulting in an alignment with a minimum sequence length of 1115 nucleotides and 1172 distinct alignment patterns in the maximum likelihood analysis. In reconstructed trees containing representatives of most recognized families within the class *Gammaproteobacteria* the OMG group separates at least into two major lineages. In the tree shown in Supplementary Figure S1 members of the clades OM60/NOR5, BD1-7, and SAR92 group together, whereas members of the OM182 and KI89A clades form a separate lineage that is not closely related to the main group. Since the first phylogenetic assessment of the OMG group by Cho and Giovannoni (2004) a large number of isolates with a close phylogenetic relationship to members of the OMG group were cultured and described as novel species. So far all of these isolates could be either affiliated to the OM60/NOR5 clade or were closely related to members of the BD1-7 and SAR92 clades, whereas no type strains or genome sequenced isolates became available belonging to the OM182 or KI89A clade which prevents a more detailed phylogenetic assessment of the latter two groups. In addition, the SAR86 clade representing another emerging group of oligotrophic marine gammaproteobacteria (Dupont et al., 2012) was not considered in this study because of its large phylogenetic distance to the targeted OMG group and a lack of cultured representatives. Most of the already classified species that cluster together with the main lineage of the OMG group were allocated to various established higher taxa within the *Gammaproteobacteria*, including *Pseudomonadaceae, Alteromonadaceae*, and *Oceanospirillaceae* (Supplementary Table S1). However, as shown in Supplementary Figure S1 the type genera of these families are only distantly related to members of the main lineage of the OMG group. Based on phylogenetic analyses using 16S rRNA sequence data a recognizable relationship to the main lineage of the OMG group could be only assumed for members of the genus *Pseudomonas*, whereas the other type genera appear to be unrelated. In Supplementary Figure S2 a subtree of the comprehensive 16S rRNA gene based tree of *Gammaproteobacteria* is presented that shows exclusively the position of sequences that are either closely related or allocated to clades of the main lineage of the OMG group. The lowest 16S rRNA gene identity values observed among members of this group were around 85% which corresponds to the minimum identity typically found in an order defined according to modern taxonomic standards (Yarza et al., 2014). Thus, we propose to define a novel taxon at order level for this deep-branching phylogenetic lineage within the class *Gammaproteobacteria* and suggest to designate it *Cellvibrionales*, because the first described type species within this group was *Cellvibrio mixtus* (Blackall et al., 1985). Note, however, that sequence identity should not be used to assign strains to taxa because more similar organisms are not necessarily more closely related (Meier-Kolthoff et al., 2014b), hence it is important to determine the minimum sequence identity for subtrees in the existing phylogenetic tree. Within this order-level lineage several monophyletic groups can be distinguished three of which contain representatives originally affiliated to the OMG group, whereas two comprise exclusively described species that were allocated to established families of *Gammaproteobacteria*. The sequence cluster represented by the OM60/NOR5 clade also comprises the genera *Congregibacter, Haliea, Halioglobus, Luminiphilus*, and *Pseudohaliea*; the BD1-7 clade forms a group together with the genera *Spongiibacter, Zhongshania, Sinobacterium*, and *Dasania*, whereas the SAR92 clade is closely related to the genus *Porticoccus* and several sequences affiliated to the C1-B045 clade that were first detected in deep-sea hydrothermal sediments (Teske et al., 2002). Clades that are mainly comprised of formally described cultured strains are represented by members of the genus *Microbulbifer* on the one hand and *Cellvibrio* species and related taxa on the other hand. It is important to note, however, that the monophyletic assemblage shown in Supplementary Figure S2 was only obtained using the neighbor-joining reconstruction method and is not supported by significant bootstrap values. In addition, the bootstrap support for the depicted phylogenetic subgroups except the OM60/NOR5 and *Microbulbifer* cluster are insignificant (below 75%) with most applied reconstruction methods, so that a formal proposal of higher order taxonomic groups within this phylogenetic branch requires further investigation.

#### Phylogeny based on RpoB proteins

**Figure 1:**
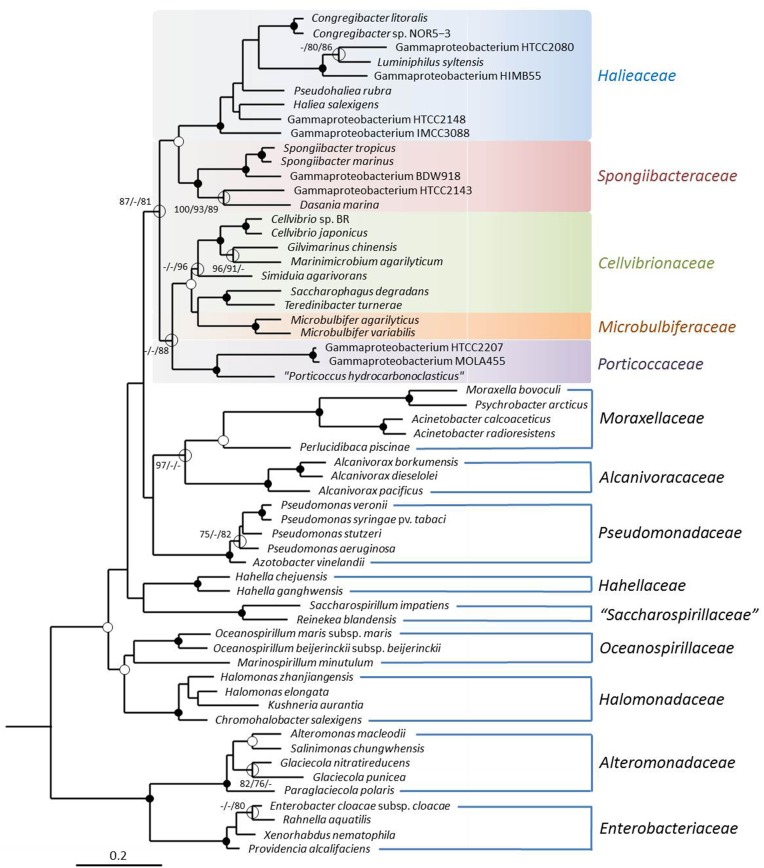
**Phylogenetic tree inferred from a dataset of complete RpoB protein sequences**. The tree topology was reconstructed with the maximum-likelihood method using the PROTCATLGF model. The RpoB sequence of *Magnetococcus marinus* MC-1^T^ was used as an outgroup (not shown). Additional trees were reconstructed using maximum parsimony and neighbor-joining methods. Support of a distinct branching by bootstrap analyses is indicated by symbols. Black dots at a distinct node indicate that bootstrap values of 95% or above (percentages of 1000 resamplings) were obtained with three different reconstruction methods, while white dots indicate that values of 95% or above were obtained with only two reconstruction methods. Hollow circles indicate that bootstrap values of 75% or above were obtained with at least one reconstruction method. In such cases the values above 75% are given from left to right for the maximum-likelihood, maximum parsimony and neighbor-joining method. The branches are scaled in terms of the expected number of substitutions per site.

In this study a dataset of 60 complete RpoB proteins derived from the available genome sequences of members of the OMG group and related gammaproteobacteria was used (Table 1). The resulting amino acid alignment comprised sequences with a minimum length of 1337 positions and 924 distinct alignment patterns in the maximum likelihood analysis. In the reconstructed phylogenetic tree shown in Figure 1 the average bootstrap support for the five identified subgroups of the proposed order *Cellvibrionales* is considerably higher than in the 16S rRNA gene based tree. To exclude the possibility that the improved stability of the RpoB tree topology is related to the reduced number of selected sequences compared to the 16S rRNA gene based tree shown in Supplementary Figure S1, trees with the same set of strains but based on 16S rRNA sequences were reconstructed. However, it turned out that the stability of the 16S rRNA gene based trees was not significantly improved using the reduced dataset compared to the more comprehensive set of sequences. In addition, the monophyly of the *Cellvibrionales* lineage was not retained using the reduced 16S rRNA gene dataset (data not shown). Hence, the improved reliability of the RpoB based phylogeny is independent of the number of used sequences and caused by the higher information content of this large conserved protein compared to the 16S rRNA. However, despite the improved stability of the phylogenetic reconstruction several subgroups of the *Cellvibrionales*, like the clade represented by *Cellvibrio* species and closely related taxa was still not resolved with high confidence values in the RpoB based tree. We used, therefore, genome-scale data to further improve the reliability of the reconstructed phylogenetic relationships.

#### Phylogeny based on genome-scale data

##### Supermatrix trees

**Figure 2:**
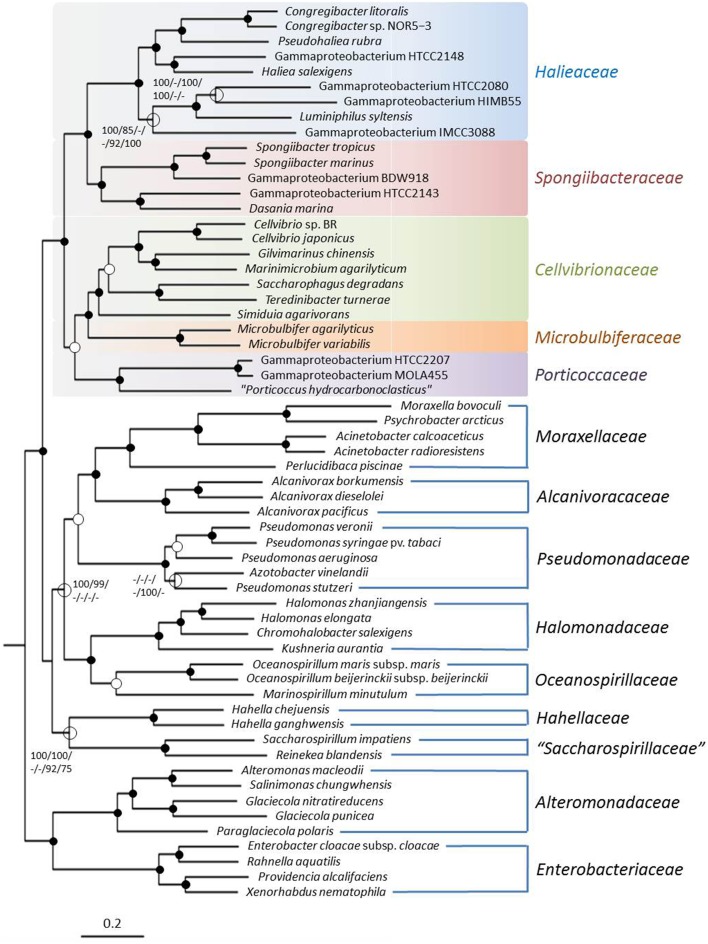
**Phylogenetic tree inferred from the 2,801,974 aligned amino acid character containing supermatrix**. The tree topology was reconstructed under the maximum likelihood criterion and was rooted with *Magnetococcus marinus* MC-1^T^ (not shown). Black dots at a distinct node indicate that bootstrap values of 95% or above (percentages of 1000 resamplings) were obtained with six different inference methods, while white dots indicate that values of 95% or above were obtained with four or five reconstruction methods. Hollow circles indicate that bootstrap values of 75% or above were obtained with at least one inference method. In such cases values above 75% are given from left to right for the ML supermatrix, MP supermatrix, ML MARE-filtered supermatrix, MP MARE-filtered supermatrix, ML core-genes matrix and MP core-genes matrix analyses. The branches are scaled in terms of the expected number of substitutions per site.

The basic supermatrix comprised 10,059 genes and 2,801,974 characters (i); a further matrix containing 928 genes and 223,650 characters was obtained after cleaning with MARE (ii). The core-genes matrix contained 341 genes and 111,505 characters (iii). For all three matrices the selected model for maximum likelihood reconstructions was PROTCATLGF; that is the LG model of amino acid evolution (Le and Gascuel, 2008) in conjunction with the CAT model approximation of rate heterogeneity (Stamatakis, 2006) and empirical amino acid frequencies. The resulting trees had a log likelihood of −53,403,618.46 (i), −9,353,599.37 (ii), and −5,308,688.73 (iii), respectively. The supermatrix maximum likelihood (ML) tree is shown in Figure 2. The best maximum-parsimony (MP) trees found had a length of 9,726,609 (i), 1,786,102 (ii), and 1,031,863 (iii) steps (not counting uninformative characters). As expected the increased number of characters in the supermatrix analyses resulted in a more stable tree topology compared to the single gene analyses with nearly maximum bootstrap support for the proposed *Cellvibrionales* lineage including the five identified subgroups. Interestingly, the close relationship of the *Cellvibrionales* to members of the *Pseudomonadaceae* that became apparent in the 16S rRNA gene based tree was not evident in the RpoB or supermatrix trees. This finding supports our proposal to separate members of the main lineage of the phylogenetically defined OMG group and related taxa from the *Pseudomonadales* or any other established order within the *Gammaproteobacteria*.

##### Gene-content analyses

**Figure 3:**
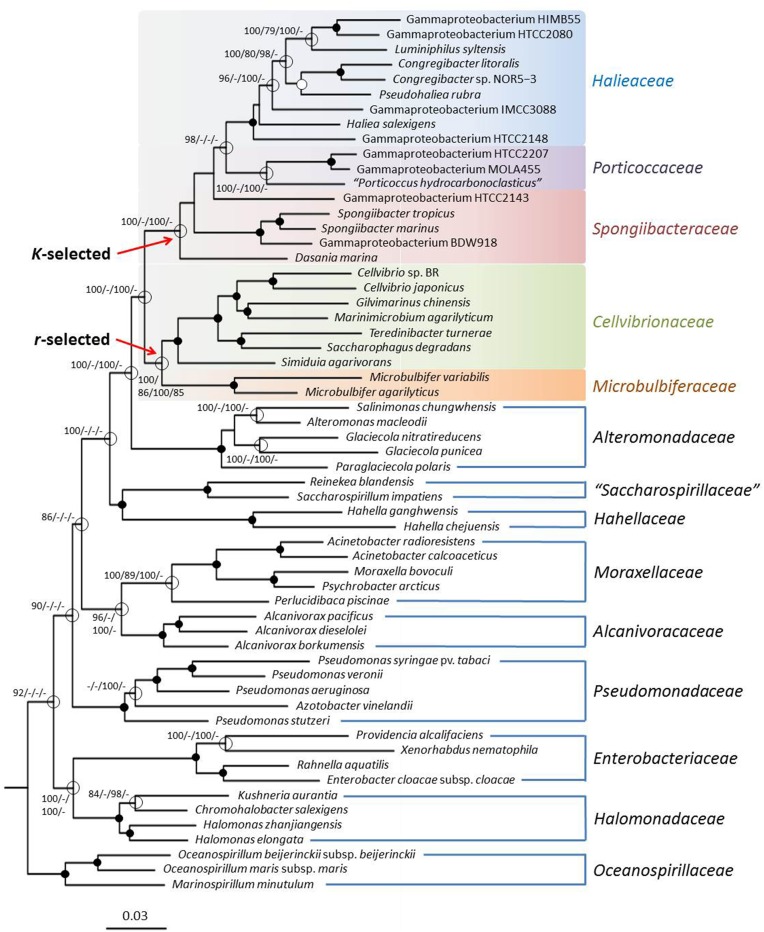
**Phylogeny inferred from a gene-content matrix**. The tree topology was reconstructed under the maximum likelihood criterion and was rooted with *Magnetococcus marinus* MC-1^T^ (not shown). Black dots at a distinct node indicate that bootstrap values of 95% or above (percentages of 1000 resamplings) were obtained with four different inference methods, while white dots indicate that values of 95% or above were obtained with three reconstruction methods. Hollow circles indicate that bootstrap values of 75% or above were obtained with at least one inference method. In such cases values above 75% are given from left to right for the ML gene-content matrix, MP gene-content matrix, ML ortholog-content matrix and MP ortholog-content matrix analyses. The estimated evolutionary origins of the two different trophic guilds within the *Cellvibrionales* are indicated by red arrows. The branches are scaled in terms of the expected number of substitutions per site.

In addition to supermatrix trees genome wide gene-content analyses can be used to obtain useful information about the relatedness of environmental bacteria. The obtained gene-content matrix contained 32,576 characters, and the resulting best trees had a log likelihood of −329,588.46 and a length of 72,280 steps, respectively. The ortholog-content matrix comprised 44,726 characters and yielded best trees with a log likelihood of −462,997.17 and a parsimony score of 102,753, respectively. The gene-content ML tree is shown in Figure 3. Although, the overall topology of the supermatrix tree was confirmed in the gene-content analyses, bootstrap support for the five subgroups within the *Cellvibrionales* was markedly reduced indicating a lower phylogenetic resolution due to a reduced number of characters or a greater effect of lateral gene transfer events on the obtained phylogenetic signal (Philippe and Douady, 2003; Kloesges et al., 2011). However, the gene-content analyses revealed another interesting aspect about this branch of the *Gammaproteobacteria*: In contrast to the sequence-matrix based phylogenetic trees a divergence between members of the originally identified OMG clades OM60/NOR5, BD1-7, and SAR92 and established taxa belonging to genera like *Cellvibrio* or *Microbulbifer* emerged. The unveiled separation of this phylogenetic branch into two major groups probably reflects two different trophic strategies, because members of the OMG clades are considered as oligotrophs, whereas representatives of the other group were mainly isolated on nutrient rich complex media and can be considered as copiotrophs. The lack of congruence in the overall branching pattern of the two different trophic groups with the phylogenetic relationships of their representatives indicate that the adaptation to oligotrophic growth conditions in the SAR92 clade on the one hand and the OM60/NOR5 and BD1-7 clades on the other hand has been probably developed from different copiotrophic ancestors.

### Correlation of phenotypic traits with the proposed evolutionary relationships

#### Chemotaxonomy

Based on the available descriptions of the cultured members of the proposed order *Cellvibrionales* chemotaxonomic data were collected and correlated with the five identified subgroups of this lineage. The obtained results along with the used literature are shown in Table 2. With only very few exceptions the major respiratory lipoquinone of representatives of the *Cellvibrionales* is ubiquinone 8 (Q8), which provides a strong argument to separate this group from the pseudomonads *in sensu stricto* which are characterized by ubiquinone 9 (Q9) as major respiratory lipoquinone (Oyaizu and Komagata, 1983; Moore et al., 2006).

**Table 2:** **Chemotaxonomic traits of genera with a proposed affiliation to the novel order *Cellvibrionales***.

**Genus**	**No. of species**	**Major quinone**	**Major CFA**	**DNA G + C content (mol%)**
***Cellvibrionaceae* fam. nov**.				
*Cellvibrio*^1,2^	8	ND	C_16:1_^a^, C_16:0_, C_18:1_	44–53
*Eionea*^3^	1	Q8	C_16:1_, C_18:1_, C_16:0_	48
*Gilvimarinus*^4^	2	Q8	C_16:1_, C_16:0_, C_18:1_	49–52
*Maricurvus*^5^	1	Q8	C_16:1_, C_18:1_, C_16:0_	49
*Marinimicrobium*^6^	3	Q8	C_16:0_, C_19:0_ cyclo, C_16:1_	57–58
*Pseudoteredinibacter*^7^	1	Q9	C_16:1_, C_17:1_, C_18:1_, C_16:0_	52
*Saccharophagus*^7^	1	ND	C_16:0_, C_17:0_, C_18:1_	46
*Simiduia*^8^	4	Q8	C_16:1_, C_17:1_, C_16:0_	50–56
*Teredinibacter*^7^	1	ND	C_18:1,_ C_16:0_, C_16:1_	49
*Umboniibacter*^9^	1	Q7	C_17:1_, C_16:0_, C_17:0_, C_16:1_	52
***HALIEACEAE* fam. nov**.				
*Haliea*^10^	2	Q8	C_16:1_, C_18:1_, C_17:1_	61–62
*Chromatocurvus*^10^	1	Q8	C_17:1_, C_16:1_, C_18:1_	63
*Congregibacter*^10^	1	Q8	C_16:1_, C_18:1_	56–58
*Halioglobus*^11^	2	Q8	C_16:1_, C_18:1_	59–60
*Luminiphilus*^10^	1	Q8	C_16:1_, C_16:0_	57
*Pseudohaliea*^10^	1	Q8	C_18:1_, C_16:0_, C_16:1_	66
***MICROBULBIFERACEAE* fam. nov**.				
*Microbulbifer*^12-16^	19	Q8	iso-C_15:0_, iso-C_17:1_, C_18:1_	49–62
***PORTICOCCACEAE* fam. nov**.				
*Porticoccus*^17^	1	ND	anteiso-C_15:0_, anteiso-C_17:0_	48
***SPONGIIBACTERACEAE* fam. nov**.				
*Spongiibacter*^18^	2	Q8	C_17:1_, C_18:1_, C_16:1_	58–61
*Dasania*^1^	1	Q8	C_16:1_, C_16:0_	47^b^
*Sinobacterium*^19^	1	Q8	C_16:1_, C_16:0_	59
*Zhongshania*^20^	4	Q8	C_16:1_, C_17:1_, C_16:0_	52–54

*CFA, cellular fatty acid*.

a *In the cited literature the summed feature 3 containing the fatty acids C_16:1_ ω7c and/or iso-C_15:0_ 2-OH is given. However, the presence of large amounts of the fatty acid C_15:0_ 2-OH in these bacteria is highly unlikely, so that in this table summed feature 3 was replaced with C_16:1_*.

b *The DNA G + C content value of Dasania marina was deduced from the genome sequence, the value give in the literature is 37 mol%. The superscript numbers indicate the following used references: 1, Lee et al. (2007); 2, Suarez et al. (2014); 3, Urios et al. (2011); 4, Cheng et al. (2015); 5, Iwaki et al. (2012); 6, Yoon et al. (2009); 7, Chen et al. (2011); 8, Park et al. (2014); 9, Romanenko et al. (2010); 10, Spring et al. (2013); 11, Park et al. (2012); 12, Baba et al. (2011); 13, Zhang et al. (2012); 14, Kämpfer et al. (2012); 15, Jeong et al. (2013); 16, Vashist et al. (2013); 17, Oh et al. (2010b); 18, Jang et al. (2011a); 19, Su et al. (2013); 20, Lo et al. (2014)*.

It further turned out that several phylogenetic groups within this order are characterized by distinct cellular fatty acid patterns that allow their differentiation from related clades. The clade comprising *Microbulbifer* species can be identified by the prevalence of *iso*-branched cellular fatty acids, whereas the presence of *anteiso*-branched fatty acids in significant amounts is specific for cells of *Porticoccus* species. These data clearly demonstrate that the revealed phylogenetic structure of this branch of the class *Gammaproteobacteria* can be correlated with phenotypic traits of the respective strains, so that the establishment of higher order taxa for the identified phylogenetic groups is corroborated by phenotypic data.

In contrast to the lipid patterns, the guanine plus cytosine content of genomic DNA (DNA G + C values) showed only little variation between the five different phylogenetic groups, with the highest average value found in the OM60/NOR5 clade (52–66 mol%) and the lowest median represented by the *Porticoccus*/SAR92 cluster (48–53 mol%).

#### Ecophysiology

The dichotomy in the *Cellvibrionales* lineage that becomes apparent in the gene-content analyses shown in Figure 3 could reflect an adaptation to different ecological niches that resulted in two basically different genetic inventories. Most members of the OMG clades OM60/NOR5, BD1-7, SAR92, and closely related strains that represent one monophyletic group in Figure 3 are numerically abundant in marine environments and were mainly isolated using nutrient-poor media, so that they are considered as typical oligotrophs (Cho and Giovannoni, 2004). The metabolism of these bacteria is adapted to allow growth under conditions of extreme carbon source limitation and nutrient depletion, so that many of these organisms have the potential to use alternative sources of energy for mixotrophic growth. For instance several members of the OM60/NOR5 clade encode *sox* genes for the utilization of reduced inorganic sulfur compounds and photosynthesis genes for the harvesting of light in the euphotic zone of the oceans. However, it seems that only light, but not thiosulfate, is used by members of this phylogenetic group as additional energy source and stimulates growth (Spring and Riedel, 2013; Spring, 2014). In stark contrast members of the genera *Cellvibrio, Microbulbifer* and related taxa that comprise the second group in the gene-content tree (Figure 3) are typical copiotrophic bacteria that prefer nutrient-rich conditions for growth. A further typical characteristic of many of these copiotrophic strains is a specialization on complex polysaccharides as substrates, which is not observed among the oligotrophic representatives of the OMG group (Spring et al., 2013). Especially, the capability to degrade complex carbohydrates like agarose, chitin, or cellulose that has been detected in many members of the genera *Cellvibrio, Microbulbifer, Marinimicrobium*, or *Simiduia* could provide these copiotrophic bacteria with a selective advantage that enables them to occupy distinct nutrient-rich ecological niches, like for instance marine snow. Consequently, the dichotomy depicted in the gene-content analyses reflects probably the existence of two basically different ecological guilds that emerged independent of the evolutionary relationships within this branch of the *Gammaproteobacteria*. However, it should be noted that several representatives belonging to the oligotrophic guild of the *Cellvibrionales* show an improved growth under nutrient-rich conditions (Spring and Riedel, 2013) so that we prefer to define the members of this group in terms of traditional ecology as typical *K*-strategists and the copiotrophic members as *r*-strategists. As outlined by Andrews and Harris (1986) the advantage of the latter definitions is that they are not restricted to the level of substrate concentrations allowing growth and, therefore, are more widely applicable. On the other hand, the theory of *r*- and *K*-selection was originally developed to describe the ecology of highly developed animals and thus has some shortcomings in respect to very small asexual proliferating organisms like bacteria. An alternative approach specific for prokaryotes was developed by Schmidt and colleagues who try to define both trophic strategies based on different response times to nutrient pulses. According to their hypothesis typical oligotrophic bacteria could be characterized as slow responders and copiotrophic bacteria as rapid responders, which would be reflected in the number of encoded rRNA operon copies and the translational power of the cell (Klappenbach et al., 2000; Dethlefsen and Schmidt, 2007). Both ecological concepts can be applied to marine microbial communities resulting in the distinction of two major trophic strategies that may be related to corresponding super-niches (Polz et al., 2006; Yooseph et al., 2010; Gifford et al., 2012): In summary, typical *r*-strategists (most copiotrophs) are characterized by short periods of fast growth that alternate with long periods of non-growth usually resulting in a low abundance in the environment interrupted by occasional blooms. These bacteria prefer nutrient-rich niches (e.g., detritus) and have a metabolism of low efficiency but high plasticity. In contrast, typical *K*-strategists (most oligotrophs) are highly abundant and have long phases of continuous growth characterized by a constant low growth rate. Niches occupied by these bacteria are usually poor in nutrients leading to substrate limitation and utilization of alternative energy sources. Their metabolism is highly efficient and specialized, but has a low flexibility.

**Figure 4:**
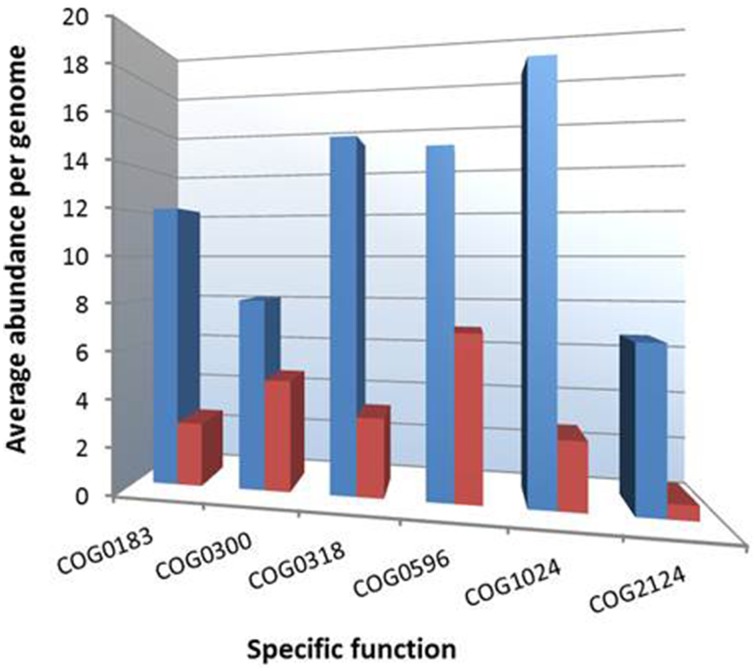
**Overrepresentation of specific COGs among genomes of presumed *K*-strategists within the proposed order *Cellvibrionales***. Blue and red bars symbolize the average abundance of specific COG categories in the genomes of 17 *K*-selected and nine *r*-selected strains, respectively. The function of the indicated COGs is as follows: COG0183, acetyl-CoA acetyltransferase; COG0300, short-chain dehydrogenases of various substrate specificities; COG0318, acyl-CoA synthetases (AMP-forming)/AMP-acid ligases II; COG0596, predicted hydrolases or acyltransferases (alpha/beta hydrolase superfamily); COG1024, enoyl-CoA hydratase/carnithine racemase; COG2124, cytochrome P450.

It was previously suggested using two distinct species of marine bacteria, one paradigmatic for a copiotrophic and the other for an oligotrophic life style, that the trophic strategies of microorganisms can be inferred from their genome sequences (Lauro et al., 2009). The authors of this study revealed that the genomes of copiotrophic species were often characterized by the overrepresentation of genes associated with the cluster of orthologous group (COG) categories motility (N), defense mechanisms (V), signal transduction mechanisms (T), and transcription (K). In contrast, oligotrophic species were enriched in COGs involved in lipid transport and metabolism (functional category I) and secondary metabolites biosynthesis, transport and catabolism (functional category Q). Accordingly, we performed a COG analysis of both sets of genomes that correspond to distinct ecological guilds in the tree shown in Figure 3 using tools of the IMG-ER server (Markowitz et al., 2009). As shown in Table 3, we could confirm the predictions made by Lauro et al. (2009) using a comprehensive dataset of whole genomes of environmental important gammaproteobacteria related to the OMG group. We applied this approach also to a set of four genomes affiliated to the distantly related gammaproteobacterial SAR86 clade. The analyzed genomes were however not obtained from pure cultures, but by metagenomic assemblies or single cell sequencing (Dupont et al., 2012). These genomes have a very small size (1.3–1.7 MBp) compared to members of the OMG group indicating a reductive evolution and genome streamlining typical for obligate oligotrophs (Giovannoni et al., 2014). Gene-content analyses revealed a low abundance of the COG categories motility (1%), signal transduction mechanisms (2%), and transcription (3%), but a relatively high prevalence of genes involved in lipid metabolism (9%) and secondary metabolites (4%), which suggests their categorization as typical *K*-strategists. In addition, we tried to identify genomic features, which allow a reliable differentiation of both trophic guilds within the *Cellvibrionales*. In Figure 4 the frequencies of several functional COGs are shown, which differ most significantly among representatives of both trophic guilds. All of these COGs were previously identified in the study of Lauro et al. (2009) and were found to be overrepresented in oligotrophs. Most of these COGs can be associated with the degradation of fatty acids, which may indicate a specialization of marine gammaproteobacterial *K*-strategists on lipids as carbon source due to their higher energy yield compared to carbohydrates (Lauro et al., 2009). In support of this result it was previously reported that a range of fatty acids with various chain lengths like acetate, propionate, butyrate or palmitate can be utilized by several representatives of the OM60/NOR5 clade (Spring et al., 2009, 2013). The largest divergence in the frequency of a distinct function was revealed for cytochrome P450 genes (COG2124) that were found to be 11 times more abundant in the genomes of postulated *K*-strategists than *r*-strategists. P450 enzymes play a role for example in the degradation and detoxification of certain soluble recalcitrant compounds that are used by some oligotrophic marine bacteria as carbon source. This is corroborated by a study that reports utilization of the xenobiotic compound nonylphenol polyethoxylate by members of the OM60/NOR5 clade (Maeda et al., 2005). In general, the combination of COG categories shown in Figure 4 could be useful for the identification of gammaproteobacteria representing *K*-strategists in marine metagenomic libraries.

**Table 3:** **Abundance of genes associated with functional COG categories in genome sequenced members of two guilds of the novel order *Cellvibrionales* that are distinguishable by their trophic strategy**.

**COG category**	**Percentage of genes associated with functional COG categories^a^**	
	***K*-strategists (17 genomes)**	***r*-Strategists (nine genomes)**
Amino acid transport and metabolism	8	7
Carbohydrate transport and metabolism	3 (−)	6 (+)
Cell cycle control, cell division, chromosome partitioning	1	1
Cell motility	2 (−)	3 (+)
Cell wall/membrane/envelope biogenesis	6	6
Coenzyme transport and metabolism	5	5
Defense mechanisms	1 (−)	2 (+)
Energy production and conversion	7	6
Inorganic ion transport and metabolism	6	6
Intracellular trafficking, secretion, and vesicular transport	3	4
Lipid transport and metabolism	7 (+)	3 (−)
Nucleotide transport and metabolism	2	2
Posttranslational modification, protein turnover, chaperones	5	5
Replication, recombination and repair	5	4
Secondary metabolites biosynthesis, transport and catabolism	4 (+)	2 (−)
Signal transduction mechanisms	3 (−)	6 (+)
Transcription	5 (−)	7 (+)
Translation, ribosomal structure and biogenesis	6	6

a *Values correspond to the average percentage of a given COG category per genome. The total for calculating individual percentage values is based on the total number of protein coding genes in the respective annotated genomes. Significant upward and downward deviations from the estimated mean values are labeled with plus or minus signs, respectively*.

In a recent study by Gifford et al. (2012) the expression patterns of a highly diverse microbial community in a coastal ocean system was studied using a metatranscriptomics approach. Interestingly, their results imply a high degree of niche diversification in seawater, an environment commonly regarded as homogenous. Several of the reported results seem to confirm predictions made by gene-content analyses for the two ecological guilds of *Cellvibrionales*. For instance, transcriptomes of three bins assigned to genome sequences of the putative *r*-strategists *Teredinibacter turnerae, Saccharophagus degradans*, and *Cellvibrio japonicus* showed a high prevalence of transcribed ribosomal protein genes (mean value of 14.5%) indicating a high translational power and growth rate, whereas bins assigned to genomes of the highly abundant predicted *K*-strategists *Luminiphilus syltensis* NOR5-1B, HTCC2080, and HTCC2207 had a significantly lower proportion of expressed ribosomal proteins (mean value of 7.6%) indicating a low growth rate. In addition, bins related to the *K*-strategists *Luminiphilus syltensis* NOR5-1B, HTCC2080, and HTCC2207 showed a relatively high expression of genes involved in mixotrophic growth reflecting their effort to continue proliferation under conditions of substrate limitation. The metatranscriptomic data indicate also a metabolism specialized on fatty acid degradation for the bins of NOR5-1B and HTCC2080. A notable discrepancy to our predictions may represent the reported high expression of genes related to motility and carbohydrate utilization allocated to the HTCC2207 bin, however it is unclear if this expression pattern reflects only specific conditions encountered in the studied site or was even selected by the used sampling method which excluded particles and surface sediments. In this context it should be also noted that in the real world a continuum of ecological strategies exists. Hence, it is unrealistic to expect a clear assignment of all environmental bacteria to one or the other extreme (e.g., Gifford et al., 2012). Nevertheless, gene-content analyses can give a first approximation of the prevailing trophic strategies of bacteria belonging to higher taxonomic ranks, which then can be examined in more detail using metatranscriptomic studies of distinct environments.

In contrast to the observed differences in the genomes of the two trophic guilds of the *Cellvibrionales* there seems to be no obvious genomic divergence between organisms preferring freshwater or marine environments, because as shown in Figure 3 soil-inhabiting members of the genus *Cellvibrio* cluster together with representatives of several genera bound to marine environments. Consequently, the salinity of the preferred habitat seems to have only little influence on the genetic inventory of these strains.

## Conclusions

In this study we demonstrate that the reconstruction of evolutionary relationships among major groups of bacteria requires the analyses of large conserved proteins like RpoB or preferably whole proteomes in order to obtain reliable results, while 16S rRNA sequence analyses alone are not sufficient. Based on our analyses the phylogenetic structure of an emerging major lineage of ecologically important *Gammaproteobacteria* was revealed. Besides supermatrix-based trees phylogenetic reconstructions based on comparative gene-content analyses were of great value for the identification of two distinct trophic guilds within a monophyletic group of marine bacteria. In this respect, a combination of genome-based classification, gene-content analyses and metatranscriptomic surveys of various environments appears to be the most promising approach to unveil the assumed correlation of the ecological functions of bacteria with their affiliation to higher taxonomic ranks (Philippot et al., 2010). An evaluation of the available phenotypic data of members of the identified phylogenetic clusters suggests the proposal of a novel order within the *Gammaproteobacteria* that comprises five novel families. Formal descriptions of the novel taxa follow below:

### Description of *Cellvibrionales* ord. nov.

*Cellvibrionales* (Cell.vi.bri.o.na'les. N.L. n. *Cellvibrio*, type genus of the order; suff. *-ales*, ending denoting an order; N.L. fem. pl. n. *Cellvibrionales*, the *Cellvibrio* order).

The order belongs to the class *Gammaproteobacteria*. Cells are gram-stain-negative, straight to bent rods, cocci or pleomorphic, non-endospore-forming, motile by means of flagella or non-motile. Aerobic, mesophilic, neutrophilic, chemoorganotrophic, respiratory type of metabolism. Most strains are positive in the oxidase test. Ubiquinone 8 (Q8) is the major respiratory lipoquinone in most species. The G + C content of the genomic DNA of most strains is in the medium range between 44 and 66 mol%. The definition of the order relies mainly on the reconstruction of phylogenetic relationships based on comparative sequence analyses of complete *rpoB* genes or whole proteomes. At present, the order contains the families *Cellvibrionaceae, Halieaceae, Microbulbiferaceae, Porticoccaceae*, and *Spongiibacteraceae*. The type genus of the order is *Cellvibrio* [Blackall et al. 1986 (Parte, 2014)].

### Description of *Cellvibrionaceae* fam. nov.

*Cellvibrionaceae* (Cell.vi.bri.o.na'ce.ae. N.L. n. *Cellvibrio*, type genus of the family; suff. *-aceae*, ending denoting a family; N.L. fem. pl. n. *Cellvibrionaceae*, the *Cellvibrio* family).

The family belongs to the order *Cellvibrionales*, class *Gammaproteobacteria*, and encompasses mainly bacteria isolated from soil, marine environments or invertebrates. The description is as for the order. In addition, the ability to use complex polysaccharides as substrates is a widespread trait among strains belonging to this family. In the cellular fatty acid patterns of most strains unsaturated fatty acids (C_16:1_, C_18:1_, or C_17:1_) are dominating. The G + C content of genomic DNA ranges from 44 to 58 mol%. At present the family comprises the genera *Cellvibrio, Eionea, Gilvimarinus, Maricurvus, Marinimicrobium, Pseudoteredinibacter, Saccharophagus, Simiduia, Teredinibacter, Umboniibacter* and the *Candidatus* Endobugula. The type genus of the family is *Cellvibrio* [Blackall et al. 1986 (Parte, 2014)].

### Description of *Halieaceae* fam. nov.

*Halieaceae* (Ha.lie.a'ce.ae. N.L. fem. n. *Haliea*, type genus of the family; suff. *-aceae*, ending to denote a family; N.L. fem. pl. n. *Halieaceae*, the *Haliea* family).

The family belongs to the order *Cellvibrionales*, class *Gammaproteobacteria*, and encompasses mainly bacteria inhabiting marine environments, especially coastal areas. The description is as for the order. In addition, some strains of this family are capable of aerobic photoheterotrophic growth using bacteriochlorophyll *a* and carotenoids for the harvesting of light. Genome analyses indicate that several strains may be able to apply proteorhodopsin for the utilization of light as additional energy source. In the cellular fatty acid patterns of most strains unsaturated fatty acids (C_16:1_ or C_18:1_) are dominating. The G + C content of genomic DNA ranges from 52 to 66 mol%. Members of this family form a stable clade in reconstructed trees based on 16S rRNA gene sequences, so that currently a reliable affiliation of novel species to this family is possible based on the comparative sequence analyses of complete 16S rRNA genes. At present the family comprises the genera *Chromatocurvus, Congregibacter, Haliea, Halioglobus, Luminiphilus*, and *Pseudohaliea* as well as some uncharacterized strains belonging to the OM60/NOR5 clade as defined by Yan et al. (2009). The type genus of the family is *Haliea* [Urios et al. 2008 (Parte, 2014)].

### Description of *Microbulbiferaceae* fam. nov.

*Microbulbiferaceae* (Mi.cro.bul.bi.fer.a'ce.ae N.L. masc. n. *Microbulbifer*, type genus of the family; suff. *-aceae*, ending to denote a family; N.L. fem. pl. n. *Microbulbiferaceae*, the Microbulbifer family).

The family belongs to the order *Cellvibrionales*, class *Gammaproteobacteria*, and encompasses mainly bacteria isolated from saline sediments or soils. The description is as for the order. In addition, resting coccoid cells are formed in stationary phase. Some strains are able to degrade complex polysaccharides. Branched chain fatty acids (iso-C_15:0_, iso-C_17:1_, or iso-C_17:0_) are dominating in cellular fatty acid patterns. The G + C content of genomic DNA ranges from 49 to 62 mol%. Members of this family form a stable clade in reconstructed trees based on 16S rRNA gene sequences, so that currently a reliable affiliation of novel species to this family is possible based on the comparative sequence analyses of complete 16S rRNA genes. The type and only genus of the family is *Microbulbifer* [González et al. 1997 (Parte, 2014)].

### Description of *Porticoccaceae* fam. nov.

*Porticoccaceae* (Por.ti.coc.ca'ce.ae. N.L. masc. n. *Porticoccus*, type genus of the family; suff. *-aceae*, ending to denote a family; N.L. fem. pl. n. *Porticoccaceae*, the *Porticoccus* family).

The family belongs to the order *Cellvibrionales*, class *Gammaproteobacteria*, and encompasses mainly bacteria inhabiting marine environments. The description is as for the order. In addition, genome analyses indicate that some strains of this family may apply proteorhodopsin for utilizing light as additional energy source. Branched chain fatty acids (anteiso-C_15:0_ or anteiso-C_17:0_) are dominating in cellular fatty acid patterns. The G + C content of genomic DNA ranges from 48 to 53 mol%. At present the family only contains the genus *Porticoccus* and some uncharacterized strains belonging to the SAR92 clade as defined by Stingl et al. (2007). The type genus of the family is *Porticoccus* [Oh et al. 2010 (Parte, 2014)].

### Description of *Spongiibacteraceae* fam. nov.

*Spongiibacteraceae* (Spon.gi.i.bac.ter.a'ce.ae. N.L. masc. n. *Spongiibacter*, type genus of the family; suff. *-aceae*, ending to denote a family; N.L. fem. pl. n. *Spongiibacteraceae*, the *Spongiibacter* family).

The family belongs to the order *Cellvibrionales*, class *Gammaproteobacteria*, and encompasses mainly bacteria inhabiting marine environments. The description is as for the order. In addition, genome analyses indicate that some strains of this family may be able to use proteorhodopsin for harvesting light as additional energy source. In the cellular fatty acid patterns of most strains unsaturated fatty acids (C_16:1_ or C_17:1_) are dominating. The G + C content of genomic DNA ranges from 47 to 61 mol%. At present the family comprises the genera *Dasania, Sinobacterium, Spongiibacter, Zhongshania, “Oceanicoccus,”* and some uncharacterized strains belonging to the BD1-7 clade as defined by Cho and Giovannoni (2004). The type genus of the family is *Spongiibacter* [Graeber et al. 2008 (Parte, 2014)].

### Conflict of interest statement

The authors declare that the research was conducted in the absence of any commercial or financial relationships that could be construed as a potential conflict of interest.

## Supplementary material

Supplementary Table S1 **Collection of 16S rRNA gene sequences representing major phylogenetic groups of the class Gammaproteobacteria**.

Supplementary Figure S1 **Phylogenetic placement of the known clades of the OMG group**. Phylogenetic analyses were based on a comprehensive dataset of almost complete 16S rRNA gene sequences representing mainly type genera within the *Gammaproteobacteria*. The sequence of *Magnetococcus marinus* MC-1^T^ was used as an outgroup (not shown). Polygons represent groups of two or more sequences. Clades of the OMG group are labeled in red. The phylogenetic lineage corresponding to the proposed order *Cellvibrionales* is shaded in blue. Quotation marks indicate names that are not yet validly published or illegitimate. The tree topology was reconstructed with the neighbor-joining method implemented in the ARB software using the correction of Felsenstein. In addition, trees were reconstructed using the maximum parsimony software of TNT and the RAxML maximum likelihood program. Support of a distinct branching by bootstrap analyses is indicated by symbols. Black dots at a distinct node indicate that bootstrap values of 95% or above (percentages of 1000 resamplings) were obtained with three different reconstruction methods, while white dots indicate that values of 95% or above were obtained with only two reconstruction methods. Hollow circles indicate that bootstrap values of 75% or above were obtained with at least one reconstruction method. In such cases the obtained values are given from left to right for the neighbor-joining, maximum-likelihood and maximum parsimony method. The scale bar represents an estimated sequence divergence.

Supplementary Figure S2 **Subtree of the comprehensive tree shown in Supplementary Figure S1**. Only the position of clones or strains affiliated to the proposed order *Cellvibrionales* is displayed. Groups of sequences identified as distinct clades are distinguished by using shades of different colors. Symbols are explained in the legend of Supplementary Figure S1.
