# Supplementary material for: A taxonomic framework for emerging groups of ecologically important marine gammaproteobacteria based on the reconstruction of evolutionary relationships using genome-scale data
Source: Front Microbiol. 2015 Apr 9;6:281. doi: 10.3389/fmicb.2015.00281 (PMC4391266; doi:10.3389/fmicb.2015.00281)
Supplement: Supplementary Table S1 — Collection of 16S rRNA gene sequences representing major phylogenetic groups of the class Gammaproteobacteria. [file TableS1.DOCX]

**Supplementary Table S1.** Collection of 16S rRNA gene sequences representing major phylogenetic groups of the class *Gammaproteobacteria*.

| Strain or clone designation | 16S rRNA gene acc. no. | Current Classification (NCBI/SILVA) |
| --- | --- | --- |
| Clades of the OMG group |  |  |
| *Chromatocurvus halotolerans* EG19^T^ | AM691086 | OM60/NOR5 |
| *Congregibacter litoralis* KT71^T^ | NZ_CM002299 | OM60/NOR5 |
| *Congregibacter* sp. NOR5-3 | ACCX01000003 | OM60/NOR5 |
| *Haliea salexigens* 3X/A02/235^T^ | AY576769 | OM60/NOR5 ^a^ |
| *Haliea mediterranea* 7SM29^T^ | FN398053 | OM60/NOR5 ^a^ |
| *Halioglobus japonicus* S1-36 ^T^ | AB602427 | Om60/NOR5 ^a^ |
| *Halioglobus pacificus* S1-72 ^T^ | AB602430 | Om60/NOR5 ^a^ |
| *Lumeniphilus syltensis* NOR5-1B^T^ | ACCY01000005 | OM60/NOR5 |
| *Pseudohaliea rubra* CM41_15a^T^ | EU161717 | OM60/NOR5 |
| Gammaproteobacterium HTCC2080 | AAVV01000015 | OM60/NOR5 |
| Gammaproteobacterium HTCC2148 | ABXQ01000070 | OM60/NOR5 |
| Gammaproteobacterium HTCC2246 | AY386337 | OM60/NOR5 |
| Gammaproteobacterium HIMB55 | AGIF02000001 | OM60/NOR5 |
| Gammaproteobacterium IMCC3088 | AEIG01000101 | OM60/NOR5 |
| Uncultured Gammaproteobacterium OM241 | U70702 | OM60/NOR5 |
| Uncultured Gammaproteobacterium ELB16-085 | DQ015807 | OM60/NOR5 |
| Uncultured Gammaproteobacterium ctg_NISAA05 | DQ396099 | OM60/NOR5 |
| Uncultured Gammaproteobacterium NEP68 | AB212806 | OM60/NOR5 |
| Uncultured Gammaproteobacterium NEP1 | AB212800 | OM60/NOR5 |
| Uncultured Gammaproteobacterium Belgica2005/10-130-14 | DQ351759 | OM60/NOR5 |
| Uncultured Gammaproteobacterium UncDee15 | AM997517 | OM60/NOR5 |
| Uncultured Gammaproteobacterium JSS S04 clone 557 | EU707309 | OM60/NOR5 |
| Uncultured Gammaproteobacterium BD2-7 | AB015537 | OM60/NOR5 |
| Uncultured Gammaproteobacterium 114ds10 | AY212565 | OM60/NOR5 |
| "*Oceanicoccus sagamiense*" PZ-5 | AB545809 | BD1-7 ^a^ |
| Gammaproteobacterium HTCC2143 | AAVT01000006 | BD1-7 |
| Uncultured Gammaproteobacterium BD1-7 | AB015519 | BD1-7 |
| Gammaproteobacterium HTCC2121 | AY386341 | SAR92 |
| Gammaproteobacterium HTCC2207 | AAPI01000014 | SAR92 |
| Gammaproteobacterium MOLA455 | AZIN01000002 | SAR92 |
| Uncultured Gammaproteobacterium ARCTIC09_G_06 | EU795093 | SAR92 |
| Uncultured Gammaproteobacterium GG101008Clone79 | JN591818 | SAR92 |
| Uncultured Gammaproteobacterium MF-Oct-12 | HQ224976 | SAR92 |
| Gammaproteobacterium HTCC2089 | AY386332 | KI89A |
| Uncultured Gammaproteobacterium KI89A | AB021704 | KI89A |
| Uncultured Gammaproteobacterium AF10-3-7_C30 | JN655243 | KI89A |
| Uncultured Gammaproteobacterium 5S29 | JF272195 | KI89A |
| Uncultured Gammaproteobacterium EK_Ca827 | JN038314 | KI89A (this study) |
| Gammaproteobacterium HTCC2178 | AY386345 | OM182 |
| Uncultured Gammaproteobacterium ARKDMS-58 | AF468261 | OM182 |
| Uncultured Gammaproteobacterium 1C226631 | EU799075 | OM182 |
| Uncultured Gammaproteobacterium T67ANG3 | AJ633982 | OM182 |
| Uncultured Gammaproteobacterium Arctic97A-11 | AF355037 | OM182 |
| Outgroup References |  |  |
| *Aeromonas hydrophila* subsp. *hydrophila* CCM 7232^T^ | DQ207728 | *Aeromonadaceae* |
| *Tolumonas auensis* TA 4r^T^ | X92889 | *Aeromonadaceae* |
| *Alcanivorax borkumensis* SK2^T^ | AM286690 | *Alcanivoracaceae* |
| *Alcanivorax pacificus* W11-5^T^ | AJGP01000039 | *Alcanivoracaceae* |
| *Alcanivorax dieselolei* B5^T^ | CP003466 | *Alcanivoracaceae* |
| *Algiphilus aromaticivorans* DG1253^T^ | DQ486493 | *Algiphilaceae* |
| *Alteromonas macleodii* ATCC 27126^T^ | CP003841 | *Alteromonadaceae* |
| *Glaciecola punicea* ACAM 611^T^ | BAET01000001 | *Alteromonadaceae* |
| *Glaciecola nitratireducens* FR1064^T^ | CP003060 | *Alteromonadaceae* |
| *Paraglaciecola polaris* LMG 21857^T^ | BAER01000001 | *Alteromonadaceae* |
| *Salinimonas chungwhensis* BH030046^T^ | AY553295 | *Alteromonadaceae* |
| *Marinimicrobium agarilyticum* M18^T^ | AY839870 | *Alteromonadaceae* |
| *Marinimicrobium koreense* M9^T^ | AY839869 | *Alteromonadaceae* |
| *Marinimicrobium locisalis* ISL-43^T^ | EU874388 | *Alteromonadaceae* |
| *Microbulbifer agarilyticus* S89 | AFPJ01000040 | *Alteromonadacea* |
| *Microbulbifer celer* ISL-39^T^ | EF486352 | *Alteromonadaceae* |
| *Microbulbifer chitinilyticus* ABABA 212^T^ | AB500894 | *Alteromonadaceae* |
| *Microbulbifer donghaiensis* CN85^T^ | EU365694 | *Alteromonadaceae* |
| *Microbulbifer elongatus* DSM 6810^T^ | AF500006 | *Alteromonadaceae* |
| *Microbulbifer epialgicus* F-104^T^ | AB266054 | *Alteromonadaceae* |
| *Microbulbifer gwangyangensis* GY2^T^ | JF751045 | *Alteromonadaceae* |
| *Microbulbifer halophilus* YIM 91118^T^ | EF674853 | *Alteromonadaceae* |
| *Microbulbifer hydrolyticus* IRE-31^T^ | U58338 | *Alteromonadaceae* |
| *Microbulbifer mangrovi* DD-13^T^ | HQ424446 | *Alteromonadaceae* |
| *Microbulbifer marinus* Y215^T^ | GQ262812 | *Alteromonadaceae* |
| *Microbulbifer maritimus* TF-17^T^ | AY377986 | *Alteromonadaceae* |
| *Microbulbifer okinawensis* ABABA 23^T^ | AB500893 | *Alteromonadaceae* |
| *Microbulbifer pacificus* SPO729^T^ | DQ993341 | *Alteromonadaceae* |
| *Microbulbifer salipaludis* SM-1^T^ | AF479688 | *Alteromonadaceae* |
| *Microbulbifer taiwanensis* CC-LN1-12^T^ | FR822983 | *Alteromonadaceae* |
| *Microbulbifer thermotolerans* JAMB A94^T^ | AB124836 | *Alteromonadaceae* |
| *Microbulbifer variabilis* Ni-2088^T^ | AB167354 | *Alteromonadaceae* |
| *Microbulbifer yueqingensis* Y226^T^ | GQ262813 | *Alteromonadaceae* |
| *Saccharophagus degradans* 2-40^T^ | CP000282 | *Alteromonadaceae* |
| *Candidatus* Endobugula glebosa | AY532642 | *Alteromonadales* |
| *Candidatus* Endobugula sertula BnSP | AF006606 | *Alteromonadales* |
| *Eionea nigra* 17X/A02/237^T^ | AY576771 | *Alteromonadales* |
| *Gilvimarinus chinensis* QM42^T^ | DQ822530 | *Alteromonadales* |
| *Maricurvus nonylphenolicus* KU41E^T^ | AB626730 | *Alteromonadales* |
| *Pseudoteredinibacter isoporae* SW-11^T^ | FJ347760 | *Alteromonadales* |
| *Teredinibacter turnerae* T7902^T^ | AY028398 | *Alteromonadales* |
| *Cardiobacterium hominis* ATCC 15826^T^ | M35014 | *Cardiobacteriaceae* |
| *Suttonella indologenes* ATCC 2586^T^ | M35015 | *Cardiobacteriaceae* |
| *Celerinatantimonas diazotrophica* S-G2-2^T^ | DQ913890 | *Celerinatantimonadaceae* |
| *Chromatium okenii* DSM 169^T^ | AJ223234 | *Chromatiaceae* |
| *Thiococcus pfennigii* 4250^T^ | Y12373 | *Chromatiaceae* |
| *Colwellia psychrerythraea* ATCC 27364^T^ | AB011364 | *Colwellaceae* |
| *Thalassomonas viridans* XOM5^T^ | AJ294747 | *Colwellaceae* |
| *Coxiella burnetii* ATCC VR-615^T^ | HM208383 | *Coxiellaceae* |
| *Ectothiorhodospira mobilis* DSM 237^T^ | X93481 | *Ectothiorhodospiraceae* |
| *Natronocella acetinitrilica* ANL 6-2^T^ | EF103128 | *Ectothiorhodospiraceae* |
| *Escherichia coli* ATCC 11775^T^ | X80725 | *Enterobacteriaceae* |
| *Enterobacter cloacae* subsp. *cloacae*  ATCC 13047^T^ | CP001918 | *Enterobacteriaceae* |
| *Rahnella aquatilis* CIP 78.65^T^ | CP003244 | *Enterobacteriaceae* |
| *Xenorhabdus nematophila* ATCC 19061^T^ | FN667742 | *Enterobacteriaceae* |
| *Providencia alcalifaciens* DSM 30120^T^ | ABXW01000071 | *Enterobacteriaceae* |
| *Buchnera aphidicola (Schizaphis graminum)* | AE013218 | *Enterobacteriaceae* |
| *Ferrimonas balearica* PAT^T^ | X93021 | *Ferrimonadaceae* |
| *Ferrimonas futtsuensis* FUT3661^T^ | AB245515 | *Ferrimonadaceae* |
| *Francisella tularensis* subsp. *tularensis* lvs^T^ | Z21931 | *Francisellaceae* |
| *Francisella halioticida* Shimane-1^T^ | JF290376 | *Francisellaceae* |
| *Granulosicoccus antarcticus* IMCC3135^T^ | EF495228 | *Granulosicoccaceae* |
| *Hahella chejuensis* KCTC 2396^T^ | CP000155 | *Hahellaceae* |
| *Hahella ganghwensis* FR1050^T^ | AY676463 | *Hahellaceae* |
| *Halomonas elongata* DSM 2581^T^ | FN869568 | *Halomonadaceae* |
| *Halomonas zhanjiangensis* JSM 078169^T^ | FJ429198 | *Halomonadaceae* |
| *Chromohalobacter salexigens* DSM 3043^T^ | CP000285 | *Halomonadaceae* |
| *Kushneria aurantia* A10^T^ | AM941746 | *Halomonadaceae* |
| *Halothiobacillus neapolitanus* CIP 104769^T^ | JN175334 | *Halothiobacillaceae* |
| *Thiovirga sulfuroxydans* SO07^T^ | AB118236 | *Halothiobacillaceae* |
| *Idiomarina abyssalis* KM227^T^ | AF052740 | *Idiomarinaceae* |
| *Aliidiomarina taiwanensis* AIT1^T^ | HQ537484 | *Idiomarinaceae* |
| *Legionella pneumophila* subsp. *pneumophila* Philadelphia 1^T^ | AE017354 | *Legionellaceae* |
| *Legionella tunisiensis* LegM^T^ | JF779686 | *Legionellaceae* |
| *Litoricola lipolytica* IMCC1097^T^ | EF176580 | *Litoricolaceae* |
| *Litoricola marina* IMCC2782^T^ | FJ943234 | *Litoricolaceae* |
| *Methylococcus capsulatus* Texas^T^ | AJ563935 | *Methylococcaceae* |
| *Methylocaldum szegediense* OR2^T^ | U89300 | *Methylococcaceae* |
| *Methylothermus thermalis* MYHT^T^ | AY829009 | *Methylothermaceae* |
| *Methylohalobius crimeensis* 10Ki^T^ | AJ581837 | *Methylothermaceae* |
| *Moraxella lacunata* ATCC 17967^T^ | AF005160 | *Moraxellaceae* |
| *Moraxella bovoculi* 237^T^ | DQ153089 | *Moraxellaceae* |
| *Psychrobacter arcticus* 273-4^T^ | CP000082 | *Moraxellaceae* |
| *Acinetobacter calcoaceticus* DSM 30006^T^ | AIEC01000170 | *Moraxellaceae* |
| *Acinetobacter radioresistens* DSM 6976^T^ | X81666 | *Moraxellaceae* |
| *Perlucidibaca piscinae* IMCC1704^T^ | DQ664237 | *Moraxellacea* |
| *Moritella marina* ATCC 15381^T^ | AB038033 | *Moritellaceae* |
| *Paramoritella alkaliphila* A3F-7^T^ | AB364966 | *Moritellaceae* |
| *Oceanospirillum linum* NBRC 15448^T^ | AB680860 | *Oceanospirillaceae* |
| *Oceanospirillum maris* subsp. *maris* ATCC 27509^T^ | AB006771 | *Oceanospirillaceae* |
| *Oceanospirillum beijerinckii* subsp. *beijerinckii* IFO 15445^T^ | AB006760 | *Oceanospirillaceae* |
| *Marinospirillum minutulum* ATCC 19193^T^ | AB006769 | *Oceanospirillaceae* |
| *Sinobacterium caligoides* SCSWE24^T^ | HQ686140 | *Oceanospirillaceae* |
| *Orbus hercynius* CN3^T^ | FJ612598 | *Orbaceae* |
| *Gilliamella apicola* wkB1^T^ | JQ936674 | *Orbaceae* |
| *Pasteurella multocida* subsp. *multocida* CCUG 17976^T^ | AF294410 | *Pasteurellaceae* |
| *Actinobacillus lignieresii* NCTC 4189^T^ | AY362892 | *Pasteurellaceae* |
| *Aggregatibacter actinomycetemcomitans* ATCC 33384^T^ | M75039 | *Pasteurellaceae* |
| *Piscirickettsia salmonis* LF-89^T^ | U36941 | *Piscirickettsiaceae* |
| *Pseudoalteromonas haloplanktis* ATCC 14393^T^ | X67024 | *Pseudoalteromonadaceae* |
| *Psychrosphaera saromensis* SA4-48^T^ | AB545807 | *Pseudoalteromonadaceae* |
| *Pseudomonas aeruginosa* PAO1 | AE004091 | *Pseudomonadaceae* |
| *Pseudomonas sutzeri* ATCC 17588^T^ | CP002881 | *Pseudomonadaceae* |
| *Pseudomonas syringae* pv. *tabaci* ATCC 11528 | AEAP01000578 | *Pseudomonadaceae* |
| *Pseudomonas veronii* 1YdBTEX2 | AOUH01000021 | *Pseudomonadaceae* |
| *Azotobacter vinelandii* ATCC BAA-1303 | CP001157 | *Pseudomonadaceae* |
| *Cellvibrio diazotrophicus* E50^T^ | JQ922426 | *Pseudomonadaceae* |
| *Cellvibrio fibrivorans* R-4079^T^ | AJ289164 | *Pseudomonadaceae* |
| *Cellvibrio fulvus* NCIMB 8634^T^ | AF448514 | *Pseudomonadaceae* |
| *Cellvibrio gandavensis* R-4069^T^ | AJ289162 | *Pseudomonadaceae* |
| *Cellvibrio japonicus* Ueda107^T^ | CP000934 | *Pseudomonadaceae* |
| *Cellvibrio mixtus* subsp. *mixtus* ACM 2601^T^ | AF448515 | *Pseudomonadaceae* |
| *Cellvibrio ostraviensis* LMG 19434^T^ | AJ493583 | *Pseudomonadaceae* |
| *Cellvibrio* sp. BR | AICM01000025 | *Pseudomonadaceae* |
| *Cellvibrio vulgaris* NCIMB 8633^T^ | AF448513 | *Pseudomonadaceae* |
| *Dasania marina* KOPRI 20902^T^ | AY771747 | *Pseudomonadales* |
| *Psychromonas antarctica* star-1^T^ | Y14697 | *Psychromonadaceae* |
| *Psychromonas agarivorans* J42-3A^T^ | AB374544 | *Psychromonadaceae* |
| *Saccharospirillum impatiens* EL-105^T^ | AJ315983 | *"Saccharospirillaceae"* |
| *Reinekea blandensis* MED297^T^ | AAOE01000002 | *"Saccharospirillaceae"* |
| *Salinisphaera shabanensis* E1L3A^T^ | AJ421425 | *Salinisphaeraceae* |
| *Salinisphaera japonica* YTM-1^T^ | AB735546 | *Salinisphaeraceae* |
| *Shewanella putrefaciens* LMG 26268^T^ | X81623 | *Shewanellaceae* |
| *Shewanella fidelis* KMM 3582^T^ | AF420312 | *Shewanellaceae* |
| *Solimonas soli* DCY12^T^ | EF067861 | *Sinobacteraceae* |
| *Nevskia ramosa* Soe1^T^ | AJ001010 | *Sinobacteraceae* |
| *Succinivibrio dextrinosolvens* DSM 3072^T^ | Y17600 | *Succinivibrionaceae* |
| *Ruminobacter amylophilus* DSM 1361^T^ | Y15992 | *Succinivibrionaceae* |
| *Thioalkalispira microaerophila* ALEN 1^T^ | AF481118 | *Thioalkalispiraceae* |
| *Thiothrix nivea* JP2^T^ | L40993 | *“Thiotrichaceae”* |
| *Thiothrix eikelboomii* AP3^T^ | AB042819 | *“Thiotrichaceae”* |
| *Vibrio cholerae* CECT 514 ^T^ | X76337 | *Vibrionaceae* |
| *Photobacterium phosphoreum* ATCC 11040^T^ | D25310 | *Vibrionaceae* |
| *Xanthomonas campestris* ATCC 33913^T^ | AE008922 | *Xanthomonadaceae* |
| *Arenimonas donghaensis* HO3-R19^T^ | DQ411038 | *Xanthomonadaceae* |
| Gammaproteobacterium BDW918 | AJMK01000058 | Gammaproteobacteria |
| Gammaproteobacterium IMCC2136 | EF468720 | Gammaproteobacteria |
| *Porticoccus litoralis* IMCC2115^T^ | EF468719 | Gammaproteobacteria |
| "*Porticoccus hydrocarbonoclasticus*" MCTG13d | JN088732 | Gammaproteobacteria |
| *Simiduia agarivorans* DSM 21679^T^ | CP003746 | Gammaproteobacteria |
| *Simiduia areninigrae* M2-5^T^ | GQ872422 | Gammaproteobacteria |
| *Simiduia curdlanivorans* DMCK3-4 ^T^ | KJ569530 | Gammaproteobacteria |
| *Simiduia litorea* KMM 9504 ^T^ | AB894237 | Gammaproteobacteria |
| *Spongiibacter marinus* (= *Melitea salexigens*) 5IX/A01/131 | AY576729 | Gammaproteobacteria |
| *Spongiibacter tropicus* CL-CB221^T^ | EF988653 | Gammaproteobacteria |
| *Umboniibacter marinipuniceus* KMM 3891^T^ | AB467279 | Gammaproteobacteria |
| *Zhongshania antarctica* ZS5-23^T^ | FJ889619 | Gammaproteobacteria |
| *Zhongshania aliphaticivorans* SM-2^T^ | KF982857 | Gammaproteobacteria |
| *Zhongshania borealis* CL-AS9^T^ | HQ199599 | Gammaproteobacteria |
| *Zhongshania guokunii* ZS6-22^T^ | FJ889678 | Gammaproteobacteria |
| Uncultured Gammaproteobacterium DH-1595 | AM292385 | C1-B045 clade |
| Uncultured Gammaproteobacterium 85-7-01 | JN019003 | C1-B045 clade |
| Uncultured Gammaproteobacterium 15 T12d+oil | FM242470 | C1-B045 clade |
| Uncultured Gammaproteobacterium CM38H2 | AM936497 | C1-B045 clade |
| Uncultured Gammaproteobacterium CM37D6 | AM936400 | C1-B045 clade |
| Uncultured Gammaproteobacterium AMDB12 | AM935570 | C1-B045 clade |
| Root |  |  |
| *Magnetococcus marinus* MC-1^T^ | CP000471 | *Magnetococcaceae* |

The indicated classification of type strains is based on the NCBI taxonomy server, whereas the ARB SILVA database was consulted to identify the phylogenetic affiliation of uncultured strains by using the SINA tool (v. 1.2.11). The superscript T after a strain designation indicates a type strain.
^a^ The given phylogenetic affiliation is based on the following reference: Spring S, Riedel T, Spröer C, Yan S, Harder J, Fuchs BM: Taxonomy and evolution of bacteriochlorophyll *a*-containing members of the OM60/NOR5 clade of marine gammaproteobacteria: Description of *Luminiphilus syltensis* gen. nov., sp. nov., reclassification of *Haliea rubra* as *Pseudohaliea rubra* gen. nov., comb. nov., and emendation of *Chromatocurvus halotolerans*. BMC Microbiol 2013, **13**:118.
