# Supplementary figures and images for: A taxonomic framework for emerging groups of ecologically important marine gammaproteobacteria based on the reconstruction of evolutionary relationships using genome-scale data
Source: Front Microbiol. 2015 Apr 9;6:281. doi: 10.3389/fmicb.2015.00281 (PMC4391266; doi:10.3389/fmicb.2015.00281)

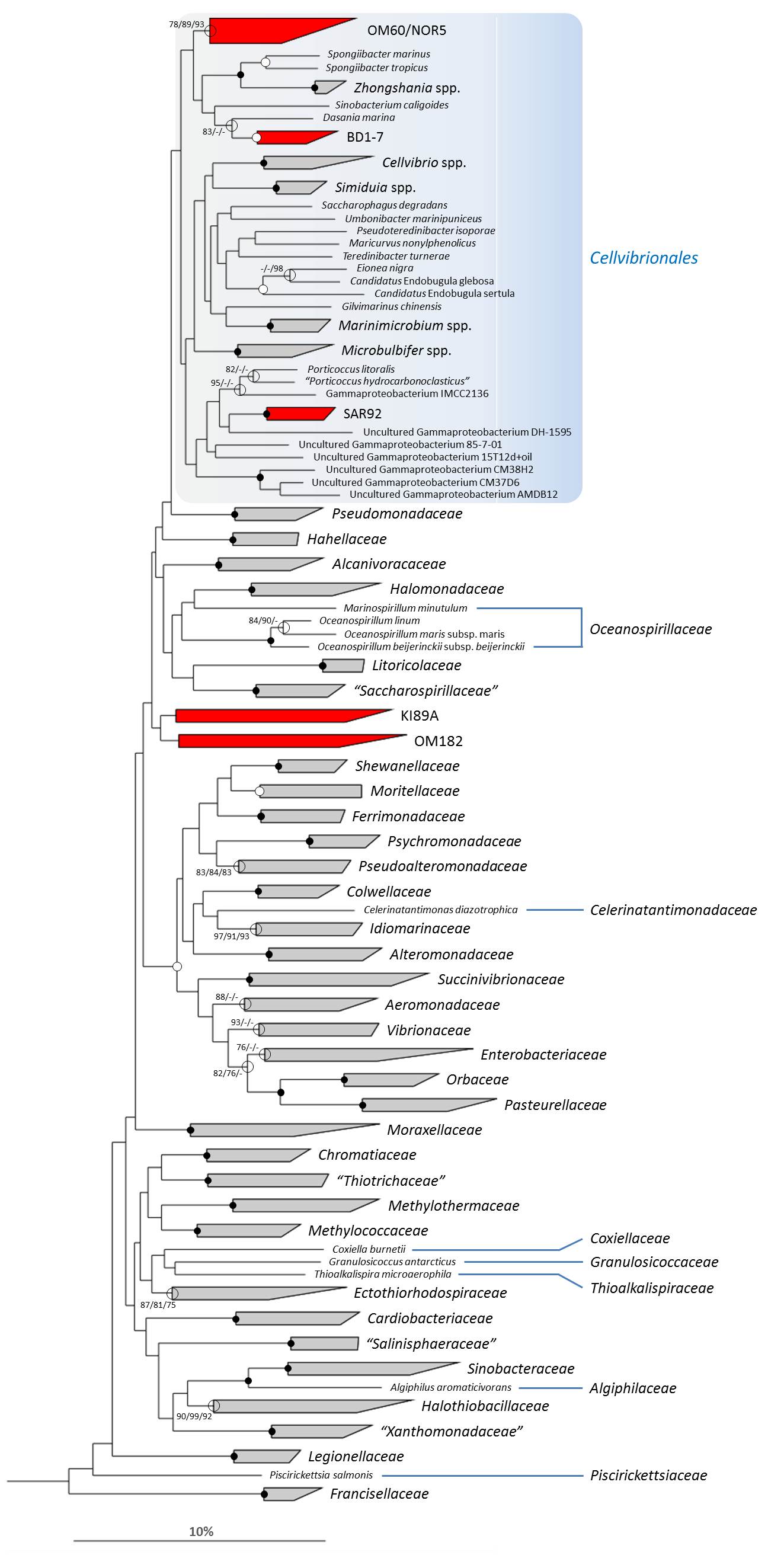

Supplement: Supplementary Figure S1 — Phylogenetic placement of the known clades of the OMG group. Phylogenetic analyses were based on a comprehensive dataset of almost complete 16S rRNA gene sequences representing mainly type genera within the Gammaproteobacteria. The sequence of Magnetococcus marinus MC-1T was used as an outgroup (not shown). Polygons represent groups of two or more sequences. Clades of the OMG group are labeled in red. The phylogenetic lineage corresponding to the proposed order Cellvibrionales is shaded in blue. Quotation marks indicate names that are not yet validly published or illegitimate. The tree topology was reconstructed with the neighbor-joining method implemented in the ARB software using the correction of Felsenstein. In addition, trees were reconstructed using the maximum parsimony software of TNT and the RAxML maximum likelihood program. Support of a distinct branching by bootstrap analyses is indicated by symbols. Black dots at a distinct node indicate that bootstrap values of 95% or above (percentages of 1000 resamplings) were obtained with three different reconstruction methods, while white dots indicate that values of 95% or above were obtained with only two reconstruction methods. Hollow circles indicate that bootstrap values of 75% or above were obtained with at least one reconstruction method. In such cases the obtained values are given from left to right for the neighbor-joining, maximum-likelihood and maximum parsimony method. The scale bar represents an estimated sequence divergence. [file FigureS1.JPEG]

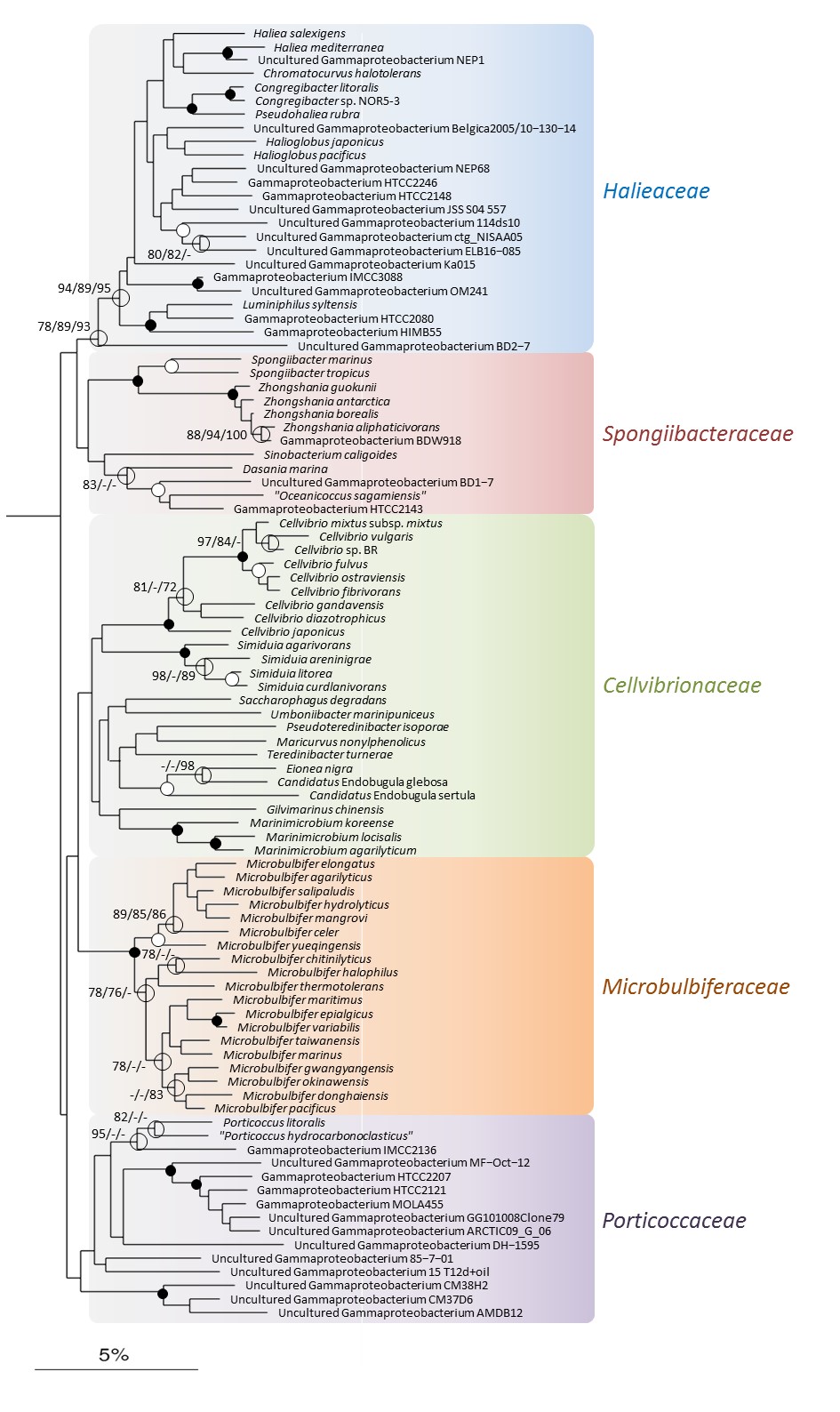

Supplement: Supplementary Figure S2 — Subtree of the comprehensive tree shown in Supplementary Figure S1. Only the position of clones or strains affiliated to the proposed order Cellvibrionales is displayed. Groups of sequences identified as distinct clades are distinguished by using shades of different colors. Symbols are explained in the legend of Supplementary Figure S1. [file FigureS2.JPEG]
